# Associations Between E-cigarette Use and E-cigarette Flavors With Cigarette Smoking Quit Attempts and Quit Success: Evidence From a U.S. Large, Nationally Representative 2018–2019 Survey

**DOI:** 10.1093/ntr/ntac241

**Published:** 2022-10-17

**Authors:** Yoonseo Mok, Jihyoun Jeon, David T Levy, Rafael Meza

**Affiliations:** Department of Integrative Oncology, BC Cancer Research Institute, Vancouver, BC, Canada; Department of Epidemiology, University of Michigan, Ann Arbor, MI, USA; Department of Epidemiology, University of Michigan, Ann Arbor, MI, USA; Department of Oncology, Georgetown University, Washington, DC, USA; Department of Integrative Oncology, BC Cancer Research Institute, Vancouver, BC, Canada; Department of Epidemiology, University of Michigan, Ann Arbor, MI, USA

## Abstract

**Introduction:**

Although many studies have examined the association between e-cigarette use and smoking cessation, fewer have considered the impact of e-cigarette flavors on cessation outcomes. This study extends previous studies by examining the effects of e-cigarette use and e-cigarette flavors on quit attempts and quit success of smoking.

**Aims and Methods:**

We used data from the 2018–2019 Tobacco Use Supplement-Current Population Survey (TUS-CPS) survey. Multivariate logistic regression analyses were used to investigate the associations between flavored e-cigarette use with quit attempts and quit success of smoking among individuals who smoked 12 months ago. Two current e-cigarette use definitions were used in these logistic regression analyses; currently use every day or some days versus 20+ days in the past 30 days.

**Results:**

Compared to those not using e-cigarettes, current every day or someday e-cigarette use with all nontobacco flavors had an adjusted odds ratio (AOR) of 2.9 (95% CI: 2.4 to 3.5) for quit attempts and 1.7 (95% CI: 1.3 to 2.2) for quit success. 20+ days e-cigarette use with flavors had stronger associations with quit attempts (AOR = 4.2, 95% CI: 3.1 to 5.5) and quit success (AOR = 4.0, 95% CI: 2.9 to 5.4). E-cigarette users with nontobacco flavors were more likely to succeed in quitting compared to those exclusively using non-flavored or tobacco-flavored e-cigarettes. Menthol or mint flavor users had slightly higher odds of quit attempts and success than users of other nontobacco flavors.

**Conclusions:**

E-cigarette use is positively associated with both making smoking quit attempts and quit success. Those using flavored e-cigarettes, particularly menthol or mint, are more likely to quit successfully.

**Implications:**

E-cigarette use is positively associated with both making a quit attempt and quit success, and those using flavored e-cigarettes are more likely to successfully quit smoking, with no statistically significant differences between the use of menthol or mint-flavored e-cigarettes versus the use of other nontobacco flavored products. This suggests that the potential for e-cigarettes to help people who currently smoke quit could be maintained with the availability of menthol or mint-flavored e-cigarettes, even if other nontobacco flavored products, which are associated with e-cigarette use among youth, were removed from the market.

## Introduction

E-cigarettes were first introduced to the US market in 2006, and their use has grown, resulting in an ongoing debate on their safety and regulation.^1^ Most e-cigarette users, particularly at older ages, currently smoke and use e-cigarettes either to quit smoking or just recreationally with no intention to quit, or formerly smoked.^2^ Some argue that dual use of cigarettes and e-cigarettes may reduce the concerns about health-related harms by those who smoke cigarettes, leading to extended smoking, and that e-cigarette use might act as a gateway to smoking, especially among youth.^3^ On the other hand, the potential of e-cigarettes to serve as a harm-reducing replacement for cigarettes or as an effective smoking cessation aid has been shown in some randomized control trials.^4^ In addition, some observational studies^5–15^ also indicate that e-cigarette use is associated with cessation behaviors and with higher rates of quit attempts and quit success, while others find conflicting results.^16–18^

Although many studies have examined the association between the frequency of e-cigarette use and smoking cessation, few studies^13,19–23^ have considered the role of e-cigarette flavors on cessation. Using ITC 2016 and 2018 surveys from Australia, Canada, England, and the United States, Li et al.^20^ found that, “sweet” flavored e-cigarette users were more likely to quit smoking between surveys compared with users of tobacco flavors. A cross-sectional study from Canada and the United States^21^ indicated that 63.1% of regular e-cigarette adult users used non-tobacco flavors, especially fruit flavors. This was especially true among former smokers who became exclusive e-cigarette users, suggesting that flavored e-cigarette use may contribute to smoking cessation. Other surveys have shown that non-tobacco flavors, such as fruit and sweet flavors, are most prevalent in both youth and adults.^20–29^ In contrast, the use of tobacco flavor is more prevalent among older e-cigarette users than in youth and young adult users, but still less prevalent than the use of sweet flavors.^21,30–35^

In this study, we extended and adapted the approach by Levy et al.^9^ to examine the role of e-cigarettes in smoking quit attempts and quit success (remaining quit from smoking for at least 3 months). Like that study, we use TUS-CPS data, which includes specific information on quit attempts in the last year for people who smoke at the time of the survey, and the time since quitting for people who previously smoked. Unlike the Levy et al. study which used the TUS-CPS 2014–2015 data, we apply the more recent TUS-CPS 2018–2019 data, which provides information on different flavors among current e-cigarette users. Thereby, we consider more recent e-cigarette use during the period when flavored pod-based devices emerged.

## Methods

### Study Population

We conducted a cross-sectional analysis of the relationship between e-cigarette use and cigarette smoking quit attempts and quit success using data from the Tobacco Use Supplement to the Current Population Survey (TUS-CPS) 2018–2019. TUS-CPS is a nationally representative survey administered as part of the U.S. Census Bureau’s Current Population Survey. The TUS-CPS 2018–2019 data includes three samples collected in July 2018, January 2019, and May 2019. We limit the analysis to self-respondents, who were asked more detailed tobacco use questions and to those who were ages 18 years and above. In addition, to analyze quitting behavior within the last year, the study population is restricted to individuals who reported smoking 12 months ago.

### Sociodemographic Variables

Participants reported sociodemographic information including gender, age (18–21, 22–25, 26–29, 30–34, 35–44, 45–64, 65 years old, and above), race (White, Black, Asian, and Other Races), Hispanic origin (Hispanic/non-Hispanic), education (less than 12th grade, high school degree, some college but no degree, college graduate, and above), family income levels (less than $19 999, $20 000–$39 999, $40 000–$74 999, $75 000, or more), marital status (never married, married-spouse present, married-spouse absent, or widowed/divorced/separated), employment status (employed/not in labor force or unemployed), residency status (metropolitan/non-metropolitan), and indoor work (yes/no).

### Cigarette Smoking Variables and Cessation Outcomes

We followed the same approach in Levy et al.,^9^ which evaluated the impact of e-cigarette use in smoking cessation using the TUS-CPS 2014–2015 data (please see Supplementary Table S1 for details on the questions used). All respondents in TUS-CPS were asked if they had smoked at least 100 cigarettes during their life and then if they now smoked cigarettes every day, some days, or not at all. Among the individuals with valid responses to these two questions (excluding “don’t know,” “refused,” and “no response”), those who currently smoke were defined as individuals who had smoked at least 100 cigarettes in their lifetime and were smoking every or some days at the time of the survey. People who currently smoke were then asked if they were “smoking cigarettes every day, some days, or not at all around this time 12 months ago.” The sample of people who smoked 12 months ago included those who currently smoke and reported smoking “every” or “some” days 12 months ago.

People who used to smoke and quit within 12 months were defined as individuals who had smoked at least 100 cigarettes in their lifetime and reported smoking 12 months ago but were not currently smoking at the time of the survey. The sample of people who smoked 12 months ago also included these individuals.

There was a total sample of 17 205 individuals who smoked 12 months ago, of which 15 049 currently smoked at the time of the survey and 2156 quit within the past 12 months. Among those, people who currently smoke with unknown quit attempts, and people who used to smoke and quit within the last 12 months but reported no cigarettes smoked 12 months ago or unknown smoking frequency 12 months ago were omitted, leaving a study sample of 16 591 (Supplementary Figure S1).

People who used to smoke 12 months ago were categorized by the number of cigarettes smoked per day (cpd): Very light (fewer than 5 cpd), light (5–14 cpd), medium (15–24 cpd), and heavy (25 or more cpd). People who reported smoking daily 12 months ago, were asked: “the average number of cigarettes smoked per day 12 months ago.” For those who smoked non-daily 12 months ago, their reported number of cigarettes per day on the days they smoked was multiplied by the number of days smoked per month and divided by 30 to measure average cpd use. An indicator variable was also included for those who classified themselves as “some days” cigarette users.

Individuals who used to smoke and quit within 12 months were grouped into categories based on their time since quit; quitting 3–12 months, 1 to less than 3 months, and less than 1 month ago.

#### Quit Attempts and Quit Success

The respondents who smoked 12 days or less in the past 30 days around this time 12 months ago were asked whether they had “tried to quit smoking completely during the past 12 months.” Respondents who smoked more than 12 days were asked whether they “stopped smoking for one day or longer because of trying to quit smoking during the past 12 months.” People who smoked 12 months ago were considered to have made a quit attempt if they answered “yes” to either of these questions.

### E-cigarette Use, E-cigarette Flavors, and Smokeless Tobacco Use

Three questions were asked about current and past e-cigarette use. After a description of e-cigarettes, participants were asked, “Have you ever used e-cigarettes even one time?” Participants were classified as ever users of e-cigarettes if they answered “yes” to this question. Ever users of e-cigarettes were then asked, “Do you now use an e-cigarette every day, some days, or not at all?.” Current e-cigarette users include those who answered “every day” or “some days.” Nonusers include those who do not currently use “every day” or “some days.” Those who responded “some days” were then asked, “On how many of the past 30-days did you use e-cigarettes?” Among current users, we differentiated those who currently used e-cigarettes at least 20 days in the past 30 days, based on results in Levy et al. An equivalent measure was also developed for smokeless tobacco (SLT) use.

We also classified e-cigarette use by flavors. E-cigarette flavors were assessed in two questions. Current e-cigarette users were asked whether they usually used flavored e-cigarettes and to indicate which of the 4 flavor categories they used (select all that apply: “Tobacco,” “Menthol or mint,” “Fruit, candy, sweets, chocolate, clove, spice, herb, or alcohol,” “Other”). Respondents indicating not using flavored e-cigarettes were further asked whether they usually used tobacco-flavored e-cigarettes. We created two e-cigarette use variables distinguishing flavor use. The first variable categorized individuals as “non-e-cigarette users,” “currently use non-flavored or exclusive tobacco-flavored e-cigarettes,” or “currently use flavored e-cigarettes.” For the second variable, we further stratified the “currently use flavored e-cigarettes” category into “currently use menthol or mint-flavored e-cigarettes regardless of using other flavors” and “currently use flavored e-cigarettes but not menthol or mint flavored.”

### Statistical Methods

For each outcome (quit attempts and quit success), chi-square analyses were conducted to test the differences within category for each of the sociodemographic variables, smoking frequency, measures of e-cigarette use, e-cigarette flavors, and SLT use.

**Figure 1.**
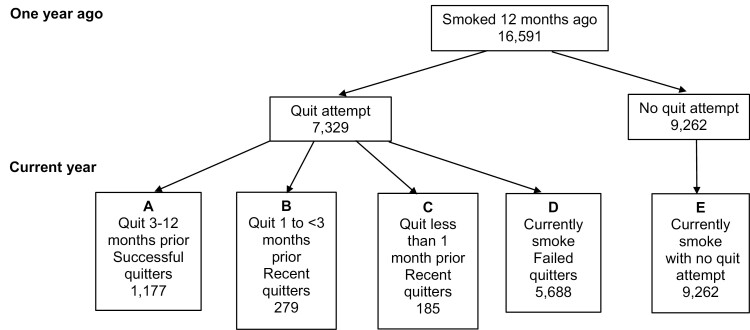
Sample design for multivariate logistic regression analysis for the association of e-cigarette use and either quit attempts or quit success.

Separate multivariate logistic regression models were fit to investigate the association between e-cigarette use and either quit attempts or quit success. In the quit attempts model, the sample included those who smoked 12 months ago, and the outcome was whether those individuals made a quit attempt in the last year. In the quit success model, the sample was limited to those having made a quit attempt, distinguishing those who failed and those who quit for longer than 3 months. Only individuals who used to smoke and who remained quit for at least 3 months were considered as quitting successfully to capture those who are more likely to remain abstinent among those who have made a quit attempt (Figure 1). Consistent with Levy et al. individuals who used to smoke for less than 3 months since quitting were removed from this analysis.^8^

We computed adjusted odds ratios (AORs) for quit attempts or quit success by including all the other covariates in the analyses. Multivariate models were fit using the “survey” package logistic regression in the R statistical program (version 4.0). All estimates accounted for self-response sample weights.

## Results

### Descriptive Statistics: Quit Attempts and Quit Success Rates

The rate (percentage %) of people who smoked 12 months ago and made a quit attempt, and the rate of quit success among those who made a quit attempt within the last 12 months are presented in Table 1. The rate of making quit attempts significantly differed (*p*-value < .05) by all individual characteristics except employment status and SLT use. Individuals with lower cigarettes per day (cpd) had a higher quit attempt rate: 1–4 (55.6%), 5–14 (46.1%), 15–24 (38.0%), 25+ (33.3%). Quit attempts significantly differed by the e-cigarette use and flavor: “currently use non-flavored or exclusive tobacco-flavored e-cigarettes” (59.0%), “currently use flavored e-cigarettes” (69.9%), “non-e-cigarette users” (42.1%).

**Table 1.** Quit Attempts Among People Who Smoked 12 Months Ago and Quit Success Among Those Making a Quit Attempt

Variable	Categories	Quit attempt model			Quit success model		
		Sample size	Quit attempts	Chi-square	Sample Size	Quit success	Chi-square
			%	(*p* value)		%	(*p* value)
Overall		16 591	44.2		6865	17.1	
Gender	Male	8368	42.6	17.83	3324	17.7	1.13
	Female	8223	45.8	(2.42 × 10^−5^)	3541	16.7	(.287)
Age	18–21y	273	57.5		141	25.5	
	22–25y	692	59.3		373	26.0	
	26–29y	1058	55.3		544	26.7	
	30–34y	1602	49.4		728	20.2	
	35–44y	3100	43.3		1263	17.7	
	45–64y	7093	41.2	196.01	2765	12.8	104.65
	≥65y	2773	40.5	(<2.2 × 10^−16^)	1051	16.8	(<2.2 × 10^−16^)
Race	White	13 768	43.3		5563	18.2	
	Black	1772	48.6		827	11.1	
	Asian	344	47.7	26.75	142	17.6	26.65
	Other Races	707	48.9	(6.66 × 10^-6^)	333	14.7	(6.97 × 10^-6^)
Hispanic	Hispanic	1169	47.4	5.14	527	19.0	1.21
	Non-Hispanic	15 422	43.9	(.023)	6338	17.0	(.271)
Education	Less than 12th grade	2335	40.9		913	11.4	
	High school degree	6479	41.9		2551	15.4	
	Some colleges, no degree	3588	47.1	50.26	1577	18.1	54.17
	College degree or higher	4189	47.0	(7.02 × 10^−11^)	1824	21.7	(1.03 × 10^−11^)
Family income	$0–$19 999	4060	46.1		1772	13.5	
	$20 000–$39 999	4456	42.6		1755	15.7	
	$40 000–$74 999	4551	43.5	12.53	1866	17.9	47.27
	$75 000 or more	3524	44.8	(.006)	1472	22.3	(3.04 × 10^−10^)
Marital status	Never Married	4656	47.7		2070	18.9	
	Married Present	5900	42.1		2326	19.1	
	Married-Spouse Absent	275	41.8	34.82	109	18.4	31.86
	Widowed/Divorced/Separated	5760	43.6	(1.33 × 10^−7^)	2360	13.6	(5.59 × 10^−7^)
Employment	Employed	9518	44.7	2.50	3970	18.8	18.79
	Not in labor force or unemployed	7073	43.5	(.114)	2895	14.8	(1.46 × 10^−5^)
Metropolitan status	Metropolitan	11 963	45.1	16.04	5069	18.0	8.67
	Non-metropolitan	4628	41.7	(6.20 × 10^−5^)	1796	14.9	(.003)
Indoor workers	No	10 559	43.0	15.44	4275	15.9	12.94
	Yes	6032	46.2	(8.53 × 10^−5^)	2590	19.3	(3.22 × 10^−4^)
Cigarettes per day 12 months ago	1–4	3197	55.6		1682	20.5	
	5–14	5923	46.1		2552	14.5	
	15–24	5668	38.0		2007	17.8	
	25+	1259	33.3	325.84	385	21.0	39.84
	Unknown CPD	544	45.0	(<2.2 × 10^−16^)	239	9.6	(4.67 × 10^−8^)
Smoking frequency	Smoked every day 12 months ago	13 226	40.4	382.50	4957	17.1	0.06
	Smoked some days 12 months ago	3365	59.1	(<2.2 × 10^−16^)	1908	17.4	(.809)
Smokeless tobacco current use	Yes	416	42.6	0.35	166	19.9	0.68
	No	15 963	44.1	(.557)	6595	17.1	(.408)
E-cigarette	Current e-cig user	1364	66.6	305.86	819	25.4	43.51
	Non-e-cig user	15 012	42.0	(<2.2 × 10^−16^)	5943	16.1	(4.22 × 10^−11^)
	Current e-cig users with non-flavored or exclusive tobacco-flavored	410	59.0		228	20.6	
	Current e-cig user with flavors	954	69.9	320.65	591	27.2	49.24
	Non-e-cig user	15 012	42.1	(<2.2 × 10^−16^)	5943	16.1	(2.03 × 10^−11^)
	Current e-cig users with non-flavored or exclusive tobacco-flavored	410	59.0		228	20.6	
	Current e-cig user with menthol or mint flavor	303	71.6		198	27.8	
	Current e-cig users with other flavors	651	69.1	321.17	393	27.0	49.30
	Non-e-cig user	15 012	42.1	(<2.2 × 10^−16^)	5943	16.1	(1.13 × 10^−10^)

The rate of quit success among those who made at least one quit attempt in the past 12 months showed significant differences (*p*-value < .05) by individual characteristics, except gender, Hispanic/non-Hispanic, smoking frequency, and SLT use. There was a clear positive gradient in quit success rates by level of income or education, i.e. individuals with higher income or education levels had a higher rate of quit success. Similar to quit attempts, the rate of quit success significantly differed by e-cigarette use and flavor: 27.2% of “current users of flavored e-cigarettes” succeeded in quitting smoking, while 20.6% and 16.1% of “current users of non-flavored or exclusive tobacco-flavored” and “non-e-cigarette users” succeeded in quitting. No differences were observed when further stratifying the e-cigarette flavor categories (quit success rate of 27.8% for current e-cigarette users with menthol or mint flavors versus 27.0% for current e-cigarette users with other non-tobacco flavors).

The characteristics of different e-cigarette use groups are presented in Supplementary Tables S2–S5. In comparison with non-e-cigarette users, a higher proportion of flavored e-cigarette users were females, young adults, White, with higher education and income, and someday smokers. In comparison with non-flavored or exclusive tobacco-flavored e-cigarette users, a higher proportion of flavored e-cigarette users were young adults and Black adults, the latter potentially reflecting the higher proportion of menthol flavor use among Black e-cigarette users.

### Logistic Regression Analysis: Quit Attempts

Table 2 presents the results of quit attempts. The first two columns show results when using the definition of e-cigarette use as “every day or some days.” The last two columns show results when using a stricter e-cigarette use definition of at least 20 of the past 30 days. In both analyses, results indicate that females and those below age 35 years, “Black” or “Other Races,” and those with higher levels of education but lower levels of income were all more likely to make a quit attempt. Individuals who smoke some days, those who smoke fewer cigarettes per day, and those who use e-cigarettes were also more likely to make a quit attempt.

**Table 2.** Logistic Regression Analysis of Having Made a Quit Attempt Among Individuals Who Smoked 12 Months Ago

		Current use						20+ days in the past 30 days					
		Model 1			Model 2			Model 1			Model 2		
		AOR	LL	UL	AOR	LL	UL	AOR	LL	UL	AOR	LL	UL
Intercept	(Intercept)	**0.7**	**0.5**	**0.9**	**0.7**	**0.5**	**0.9**	**0.7**	**0.5**	**0.9**	**0.7**	**0.5**	**0.9**
Gender (Female)	Male	**0.9**	**0.8**	**0.9**	**0.9**	**0.8**	**0.9**	**0.8**	**0.8**	**0.9**	**0.8**	**0.8**	**0.9**
Age (45–64)y	18–21y	**1.8**	**1.3**	**2.6**	**1.8**	**1.3**	**2.6**	**1.9**	**1.3**	**2.6**	**1.9**	**1.3**	**2.6**
	22–25y	**1.9**	**1.5**	**2.3**	**1.9**	**1.5**	**2.3**	**1.9**	**1.5**	**2.3**	**1.9**	**1.5**	**2.4**
	26–29y	**1.5**	**1.3**	**1.8**	**1.5**	**1.3**	**1.8**	**1.5**	**1.3**	**1.8**	**1.5**	**1.3**	**1.8**
	30–34y	**1.3**	**1.2**	**1.5**	**1.3**	**1.2**	**1.5**	**1.4**	**1.2**	**1.6**	**1.4**	**1.2**	**1.6**
	35–44y	1.0	0.9	1.2	1.0	0.9	1.2	1.0	0.9	1.2	1.1	0.9	1.2
	≥65y	1.0	0.9	1.1	1.0	0.9	1.1	1.0	0.9	1.1	1.0	0.9	1.1
Race (White)	Black	**1.2**	**1.0**	**1.4**	**1.2**	**1.0**	**1.4**	**1.2**	**1.0**	**1.3**	**1.2**	**1.0**	**1.3**
	Asian	1.0	0.8	1.3	1.0	0.8	1.3	1.0	0.8	1.3	1.0	0.8	1.3
	Other Races	**1.3**	**1.0**	**1.6**	**1.3**	**1.0**	**1.6**	**1.3**	**1.0**	**1.6**	**1.3**	**1.0**	**1.6**
Hispanic	Non-Hispanic	1.0	0.9	1.2	1.0	0.9	1.2	1.0	0.9	1.2	1.0	0.9	1.2
Education (Less than 12th grade)	High school degree	1.0	0.9	1.2	1.0	0.9	1.2	1.0	0.9	1.2	1.0	0.9	1.2
	Some college but no degree	**1.2**	**1.0**	**1.4**	**1.2**	**1.0**	**1.4**	**1.2**	**1.0**	**1.4**	**1.2**	**1.0**	**1.4**
	College degree or higher	**1.2**	**1.0**	**1.4**	**1.2**	**1.0**	**1.4**	**1.2**	**1.0**	**1.4**	**1.2**	**1.0**	**1.4**
Family income ($0-$19,999)	$20 000–$39 999	**0.8**	**0.7**	**0.9**	**0.8**	**0.7**	**0.9**	**0.8**	**0.7**	**0.9**	**0.8**	**0.7**	**0.9**
	$40 000–$74 999	**0.8**	**0.7**	**0.9**	**0.8**	**0.7**	**0.9**	**0.8**	**0.7**	**0.9**	**0.8**	**0.7**	**0.9**
	$75 000 or more	**0.9**	**0.7**	**1.0**	**0.9**	**0.7**	**1.0**	**0.8**	**0.7**	**1.0**	**0.8**	**0.7**	**1.0**
Martial status (Married-Spouse present)	Never Married	0.9	0.8	1.0	0.9	0.8	1.0	0.9	0.8	1.0	0.9	0.8	1.0
	Married-Spouse Absent	0.9	0.6	1.2	0.9	0.6	1.2	0.9	0.6	1.2	0.9	0.6	1.2
	Widowed/Divorced/Separated	1.1	1.0	1.2	1.1	1.0	1.2	1.1	1.0	1.2	1.1	1.0	1.2
Employment	Not in labor force or employed	1.0	0.9	1.2	1.0	0.9	1.2	1.0	0.9	1.2	1.0	0.9	1.2
Metropolitan status	Non-metropolitan	0.9	0.8	1.0	0.9	0.8	1.0	**0.9**	**0.8**	**1.0**	0.9	0.8	1.0
Indoor workers	Yes	1.0	0.9	1.2	1.0	0.9	1.2	1.0	0.9	1.2	1.0	0.9	1.2
Cigarettes per day 12 months ago (1–4)	5–14	1.0	0.9	1.2	1.0	0.9	1.2	1.0	0.9	1.2	1.0	0.9	1.2
	15–24	**0.8**	**0.7**	**0.9**	**0.8**	**0.7**	**0.9**	**0.8**	**0.7**	**1.0**	**0.8**	**0.7**	**1.0**
	25+	**0.7**	**0.6**	**0.8**	**0.7**	**0.6**	**0.8**	**0.7**	**0.6**	**0.9**	**0.7**	**0.6**	**0.9**
	Unknown CPD	**0.7**	**0.6**	**0.9**	**0.7**	**0.6**	**0.9**	**0.7**	**0.6**	**0.9**	**0.7**	**0.6**	**0.9**
Smoking frequency (Smoking every day)	Smoking some days	**1.8**	**1.6**	**2.1**	**1.8**	**1.6**	**2.1**	**1.9**	**1.6**	**2.1**	**1.9**	**1.6**	**2.1**
SLT use (No)	Yes	0.9	0.6	1.2	0.9	0.6	1.2	1.1	0.7	1.7	1.1	0.7	1.6
Date (July 2018)	(January 2019)	1.0	0.9	1.1	1.0	0.9	1.1	1.0	0.9	1.1	1.0	0.9	1.1
	(May 2019)	1.0	0.9	1.1	1.0	0.9	1.1	1.0	0.9	1.1	1.0	0.9	1.1
E-cigarette use (Nonusers)	Current e-cig users with non-flavored or exclusive tobacco-flavored	**2.1**	**1.6**	**2.7**	NA			**3.0**	**2.0**	**4.6**	NA		
	Current e-cig user with flavors	**2.9**	**2.4**	**3.5**				**4.2**	**3.1**	**5.5**			
	Current e-cig users with non-flavored or exclusive tobacco-flavored	NA			**2.1**	**1.6**	**2.7**	NA			**3.0**	**2.0**	**4.6**
	Current e-cig user with menthol or mint flavor				**3.0**	**2.2**	**4.2**				**5.1**	**2.9**	**8.7**
	Current e-cig users with other flavors				**2.8**	**2.2**	**3.5**				**3.8**	**2.7**	**5.3**
Model fit	Wald Statistic	16.71			16.24			15.86			15.37		

AOR = adjusted odds ratio. Statiscally significant results are highlighted in bold font.

Among e-cigarette users, two different flavor categorizations were considered. In the first, where any flavored e-cigarette use was distinguished from non-flavored or exclusive tobacco-flavored (model 1), both non-flavored or exclusive tobacco-flavored e-cigarette users (AOR 2.1, 95% CI: 1.6 to 2.7) and flavored e-cigarette users (AOR 2.9, 95% CI: 2.4 to 3.5) had significantly higher rates of quit attempts compared to non-e-cigarette users. When menthol or mint was further distinguished from other non-tobacco flavors (model 2), both flavored categories of e-cigarette users showed higher rates of quit attempts than non-flavored or exclusive tobacco-flavored e-cigarette users, although all e-cigarette use categories had statistically significant higher rates of quit attempts versus e-cigarette nonusers. Interestingly, current e-cigarette users of menthol or mint flavors had slightly higher odds of making a quit attempt (AOR 3.0, 95% CI: 2.2 to 4.2) versus current e-cigarette users of other non-tobacco flavors (AOR 2.8, 95% CI: 2.2 to 3.5), although the difference was not statistically significant (model 2).

Similar results were seen when restricting e-cigarette use to those who used at least 20 of the past 30 days, but frequent users had higher rates of quit attempts (generally AOR of 3–5 compared to AOR of 2–3) than current e-cigarette every day or someday users.

### Logistic Regression Analysis: Quit Success

Table 3 presents the results for quit success. In general, we find that those below the age of 45 years, with higher levels of education, higher levels of income, and living in metropolitan areas were more likely to succeed in quitting smoking. Current every day or someday e-cigarette users who use flavored e-cigarettes were more likely to quit smoking successfully compared to e-cigarette nonusers (model 3, AOR 1.7, 95% CI: 1.3 to 2.2). However, e-cigarette users of non-flavored or exclusive tobacco-flavored products did not have higher quit success rates versus e-cigarette nonusers (AOR 1.2, 95% CI: 0.8 to 1.8). When menthol or mint-flavored e-cigarette users were distinguished from users of e-cigarettes with other non-tobacco flavors, their likelihood of quitting smoking (AOR 1.9, 95% CI: 1.3–2.9) was slightly higher than that of e-cigarette users with other non-tobacco flavors (AOR 1.6, 95% CI: 1.2–2.2), although the difference was not statistically significant (model 4). When considering only frequent e-cigarette use (20 +days), e-cigarette users had higher rates of quit success versus nonusers regardless of flavor use (models 3 & 4).

**Table 3.** Logistic Regression Analysis of Having Made a Quit Success Among Individuals Who Smoked 12 Months Ago and Made At Least One Quit Attempt

		Current use						20+ days in past 30 days					
		Model 3			Model 4			Model 3			Model 4		
		AOR	LL	UL	AOR	LL	UL	AOR	LL	UL	AOR	LL	UL
Intercept	(Intercept)	**0.1**	**0.1**	**0.2**	**0.1**	**0.1**	**0.2**	**0.1**	**0.1**	**0.2**	**0.1**	**0.1**	**0.2**
Gender (Female)	Male	1.1	0.9	1.2	1.1	0.9	1.3	1.0	0.9	1.2	1.0	0.9	1.2
Age (45–64)y	18–21y	**1.8**	**1.1**	**3.1**	**1.8**	**1.1**	**3.1**	1.7	1.0	2.8	1.6	1.0	2.8
	22–25y	**2.5**	**1.7**	**3.5**	**2.5**	**1.7**	**3.5**	**2.3**	**1.6**	**3.3**	**2.3**	**1.6**	**3.3**
	26–29y	**2.0**	**1.5**	**2.7**	**2.0**	**1.5**	**2.7**	**1.8**	**1.4**	**2.5**	**1.8**	**1.4**	**2.5**
	30–34y	**1.5**	**1.2**	**2.0**	**1.5**	**1.2**	**2.0**	**1.5**	**1.1**	**2.0**	**1.5**	**1.1**	**2.0**
	35–44y	**1.3**	**1.0**	**1.6**	**1.3**	**1.0**	**1.6**	**1.3**	**1.0**	**1.6**	**1.3**	**1.0**	**1.6**
	≥65y	1.3	1.0	1.7	1.3	1.0	1.7	1.3	1.0	1.7	1.3	1.0	1.7
Race (White)	Black	**0.7**	**0.5**	**0.9**	**0.7**	**0.5**	**0.9**	**0.7**	**0.5**	**0.9**	**0.7**	**0.5**	**0.9**
	Asian	0.9	0.5	1.4	0.9	0.5	1.4	1.0	0.6	1.6	1.0	0.6	1.6
	Other Races	0.8	0.5	1.2	0.8	0.5	1.2	0.9	0.6	1.3	0.9	0.6	1.3
Hispanic	Non-Hispanic	1.0	0.7	1.3	1.0	0.7	1.3	0.9	0.7	1.2	0.9	0.7	1.2
Education (Less than 12th grade)	High school degree	**1.5**	**1.1**	**2.1**	**1.5**	**1.1**	**2.0**	**1.5**	**1.1**	**2.0**	**1.5**	**1.1**	**2.0**
	Some college but no degree	**1.7**	**1.3**	**2.3**	**1.7**	**1.3**	**2.3**	**1.7**	**1.2**	**2.3**	**1.7**	**1.2**	**2.3**
	College degree or higher	**2.0**	**1.4**	**2.6**	**2.0**	**1.4**	**2.6**	**1.9**	**1.4**	**2.6**	**1.9**	**1.4**	**2.6**
Family income ($0–$19 999)	$20 000–$39 999	1.1	0.9	1.5	1.1	0.9	1.5	1.2	0.9	1.5	1.2	0.9	1.5
	$40 000–$74 999	1.1	0.9	1.4	1.1	0.9	1.4	1.1	0.9	1.5	1.1	0.9	1.5
	$75 000 or more	**1.4**	**1.1**	**1.9**	**1.4**	**1.1**	**1.9**	**1.4**	**1.1**	**1.9**	**1.4**	**1.1**	**1.9**
Marital status (Married-Spouse present)	Never Married	0.8	0.7	1.0	0.8	0.7	1.0	0.8	0.7	1.0	0.8	0.7	1.0
	Married-Spouse Absent	1.1	0.6	2.2	1.1	0.6	2.2	1.1	0.6	2.2	1.1	0.6	2.2
	Widowed/Divorced/Separated	0.8	0.7	1.0	0.8	0.7	1.0	0.8	0.7	1.0	0.8	0.7	1.0
Employment	Not in labor force or employed	1.1	0.8	1.4	1.1	0.8	1.4	1.1	0.9	1.4	1.1	0.9	1.4
Metropolitan status	Non-metropolitan	**0.8**	**0.6**	**0.9**	**0.8**	**0.6**	**0.9**	**0.8**	**0.6**	**0.9**	**0.8**	**0.6**	**0.9**
Indoor workers	Yes	1.1	0.9	1.4	1.1	0.9	1.4	1.1	0.9	1.4	1.1	0.9	1.4
Cigarettes per day 12 months ago (1-4)	5–14	**0.6**	**0.5**	**0.8**	**0.6**	**0.5**	**0.8**	**0.6**	**0.5**	**0.8**	**0.6**	**0.5**	**0.8**
	15–24	0.8	0.6	1.1	0.8	0.6	1.1	0.8	0.6	1.1	0.8	0.6	1.1
	25+	1.2	0.8	1.8	1.2	0.8	1.8	1.2	0.8	1.9	1.2	0.8	1.9
	Unknown CPD	**0.4**	**0.2**	**0.8**	**0.4**	**0.2**	**0.8**	**0.4**	**0.2**	**0.8**	**0.4**	**0.2**	**0.8**
Smoking frequency (Smoking every day)	Smoking some days	0.9	0.7	1.2	0.9	0.7	1.2	0.9	0.7	1.2	0.9	0.7	1.2
SLT use (No)	Yes	0.9	0.5	1.5	0.9	0.5	1.5	1.8	0.8	3.8	1.8	0.8	3.8
Date (July 2018)	(January 2019)	**1.3**	**1.1**	**1.6**	**1.3**	**1.1**	**1.6**	**1.3**	**1.1**	**1.6**	**1.3**	**1.1**	**1.6**
	(May 2019)	**1.4**	**1.1**	**1.7**	**1.4**	**1.1**	**1.7**	**1.4**	**1.1**	**1.7**	**1.4**	**1.1**	**1.7**
E-cigarette use (Nonusers)	Current e-cig users with non-flavored or exclusive tobacco-flavored	1.2	0.8	1.8	NA			**2.6**	**1.6**	**4.3**	NA		
	Current e-cig user with flavors	**1.7**	**1.3**	**2.2**				**4.0**	**2.9**	**5.4**			
	Current e-cig users with non-flavored or exclusive tobacco-flavored	NA			1.2	0.8	1.8	NA			**2.6**	**1.6**	**4.3**
	Current e-cig user with menthol or mint flavor				**1.9**	**1.3**	**2.9**				**4.6**	**2.7**	**7.9**
	Current e-cig users with other flavors				**1.6**	**1.2**	**2.2**				**3.6**	**2.5**	**5.3**
Model fit	Wald Statistic	5.48			5.37			7.44			7.19		

AOR = adjusted odds ratio. Statiscally significant results are highlighted in bold font.

## Discussion

The results clearly indicate that those who use e-cigarettes more intensely (at least 20 of the past 30 days) and those who use flavored e-cigarettes have both a higher odds of making a quit attempt and of succeeding in quitting cigarette smoking. Current e-cigarette users of menthol or mint flavors had higher odds of making quit attempts and quit success versus current e-cigarette users of other non-tobacco flavors, although the differences were not statistically significant.

The consistency of our findings with results from randomized control trials of e-cigarettes as smoking cessation aids^4^ and with those from other observational studies strengthens the evidence that e-cigarettes can help people who smoke quit.^5–15^ In particular, our results are consistent with previous studies using the earlier TUS-CPS 2014–2015 data that reported associations of e-cigarette use, particularly frequent use, with smoking cessation.^8,9^ Our findings are also consistent with another study of TUS-CPS 2018–2019 data that found that e-cigarette use is associated with smoking cessation contemplation and preparation, with stronger associations with more frequent e-cigarette use.^13^ Studies of the nationally representative longitudinal Population Assessment of Tobacco and Health (PATH) data have also reported associations of e-cigarette use with smoking abstinence^36^ and with smoking cessation.^14,15^ In contrast, some other PATH studies have found no relationship between e-cigarette use with increased smoking cessation^16^ or reduced smoking relapse.^17,18^ Further research, particularly studies covering more recent periods with newer generation e-cigarette products, are needed to reconcile these differences.

Consistent with our results that flavored e-cigarette use is associated with increased odds of making a cigarette smoking quit attempt and quit success, using waves 1 to 4 of the PATH data, Friedman et al.^19^ found that flavored e-cigarettes use is associated with smoking cessation in adults. In addition, a cross-sectional study using data of adults from Canada and the United States^21^ also found increased odds of making a quit attempt when using flavored e-cigarettes. This study also found that non-tobacco flavors (menthol or mint, fruit, candy, or other) were most likely to be used among those who used to smoke, which is consistent with our findings of the association of flavored e-cigarette use with quit success. In contrast with these findings and ours, a recent analysis of the association of e-cigarette use with cessation behaviors in TUS-CPS 2018–2019 found no association between e-cigarettes with non-tobacco flavors and with contemplation or preparation to quit relatively to the use of tobacco-flavored e-cigarettes.^13^ A systematic review study found inconclusive evidence of the association of flavored e-cigarette use with smoking cessation.^23^ Further studies are thus needed to better understand the role that e-cigarette flavors can have in different aspects of smoking cessation and relapse among recent quitters.

Our findings indicate that e-cigarette non-tobacco flavors can be helpful for smoking cessation. Interestingly, we found no evidence of a difference in the odds of quit attempts or success between users of menthol or mint versus other non-tobacco flavors. This suggests that the potential for e-cigarettes to help people who currently smoke quit could be maintained with the availability of menthol or mint-flavored e-cigarettes, even if other nontobacco flavored products, which are associated with e-cigarette use among youth,^37,38^ were removed from the market. However, the role of e-cigarettes in supporting smoking cessation could be somewhat diminished to the extent that some potential users might prefer sweetened to menthol or mint flavors.

A U.S. longitudinal study conducted in six metropolitan areas indicated that 39.1% of e-cigarette users aged 18–34 years would continue to use e-cigarettes if e-cigarettes were restricted to only tobacco flavors, while 33.2% of them would most likely switch back to cigarettes.^28^ In a similar survey conducted in Canada, England, and the United States, when asked if currently available flavors were restricted, 28.8% of regular e-cigarette users reported that they would continue using e-cigarettes that would be available after the flavor ban, 28.3% would try to continue using e-cigarettes with banned flavors, 17.1% would return to smoking, 12.9% would stop vaping and not return to smoking, and 12.9% reported that they did not know what they would do.^39^ A discrete choice experiment of 2031 adult smokers and recent quitters suggested that a flavor ban on e-cigarettes alone would likely increase cigarette use.^26^ Other studies have reported an increase in the preference for cigarettes with flavor restrictions on e-cigarettes.^27,40^ In contrast, Kasza et al.^41^ found no statistically significant differences in the rates of cigarette smoking quit attempts among dual users as a function of e-cigarette flavor use. Similarly, Meernik et al.^42^ found that non-menthol and tobacco flavors in e-cigarettes are a primary reason for adult cigarette smokers’ e-cigarette use, but that the role of flavored e-cigarettes on smoking cessation remains unclear.

Our study has some key limitations. Like other cross-sectional association studies, our results depend on the retrospective self-reported statements about behavior in the past year rather than observed behavioral changes as in longitudinal data. Self-reported data are subject to several biases and limitations, including recall bias, a tendency of providing socially acceptable answers, inaccurate self-assessment, and differential interpretation of survey questions. No data were available to assess the levels of under or over-reporting of cigarette smoking use a year before and smoking cessation three or more months prior to the survey. Related, e-cigarette flavor categorization was based on self-reported use at the time of the survey. However, e-cigarette users may have used flavored and non-flavored products at different periods during the past year and at different points in their quit attempt or quit success process. Further longitudinal observational and randomized smoking cessation studies of e-cigarette and flavors use among people who currently smoke are thus needed to better assess their causal role in cessation outcomes. Another key limitation is that we did not distinguish e-cigarette use by device type or the nicotine strength of the liquids, which may be other key product features that influence quit attempts or quit success rates. Preliminary analyses (data not shown) evaluating the interactions of device type and flavors suggest that users of tank e-cigarettes who use flavored liquids have higher quit success rates than users of tank e-cigarettes with non-flavored or exclusive tobacco-flavored liquids. However, data was sparse when stratifying by flavor and device type of e-cigarettes, resulting in unstable interaction estimates. Future analyses will attempt to combine the TUS-CPS data with other national surveys to address small sample size limitations. Finally in this study, we defined quit success as remaining quit from smoking for at least three months based on self-reported data. TUS-CPS data does not collect biospecimens, precluding us from using biochemically verified measures of smoking abstinence.^43^

Despite these limitations, our study indicates that e-cigarette use is positively associated with both making a quit attempt and quit success, and those using flavored e-cigarettes are more likely to successfully quit smoking, with no statistically significant differences between use of menthol or mint-flavored e-cigarettes versus use of other nontobacco flavored products. Future studies should investigate the relationship of frequency of e-cigarette use with smoking quit attempts or success using longitudinal data. Randomized controlled trials evaluating the efficacy of e-cigarette use as smoking cessation aids versus other cessation interventions, comparing the relative efficacy of flavored versus nonflavored or tobacco-flavored e-cigarettes, are also critically needed.^44^ In addition, further studies investigating the joint effects of e-cigarette device type, nicotine content, and flavors on smoking cessation are needed. It will be also important to follow those who may have different patterns of e-cigarette use over time, and to consider the impact of newer versus older generations of e-cigarettes in helping smokers quit and in helping recent quitters avoid relapse.

## Supplementary Material

A Contributorship Form detailing each author’s specific involvement with this content, as well as any supplementary data, are available online at https://academic.oup.com/ntr.

ntac241_suppl_Supplementary_Material

## Data Availability

Data used is publicly available for download at the TUS-CPS website https://cancercontrol.cancer.gov/brp/tcrb/tus-cps.
