# Supplementary material for: Associations Between E-cigarette Use and E-cigarette Flavors With Cigarette Smoking Quit Attempts and Quit Success: Evidence From a U.S. Large, Nationally Representative 2018–2019 Survey
Source: Nicotine Tob Res. 2022 Oct 17;25(3):541–52. doi: 10.1093/ntr/ntac241 (PMC9910159; doi:10.1093/ntr/ntac241)
Supplement: ntac241_suppl_Supplementary_Material [file ntac241_suppl_supplementary_material.docx]

**Table S1.** List of questionnaires used for cigarette smoking variables and cessation outcomes

| **Description** | **Questionnaire** |
| --- | --- |
| **Established smoking** | (Have/Has)(you/name) smoked at least 100 cigarettes in (your/his/her) entire life? 100 cigarettes = approximately 5 packs |
| **Current smoking status** | (Do/Does)(you/name) now smoke cigarettes every day, some days, or not at all? |
| **Smoking status 12 months ago** | Around this time 12 MONTHS AGO, were you smoking cigarettes every day, some days, or not at all? |
| **Everyday smokers 12 months ago** | |
| **Average number of cigarettes smoked per day 12 months ago** | Around this time 12 MONTHS AGO, on the average, about how many cigarettes did you smoke each day? FR: One pack usually equals 20 cigarettes. If converting packs to cigarettes, always verify calculation with respondent. |
| **Someday smokers 12 months ago** | |
| **Number of days smoked 12 months ago (B10a)** | Around this time 12 MONTHS AGO, on how many of the 30 days in the month did you smoke cigarettes? FR: Enter 0 for none. |
| **Average number of cigarettes smoked per day 12 months ago** | On the average, on those (Fill entry B10a) days, how many cigarettes did you usually smoke each day? |
| **Smoked 12 days or less in the past 30-days around this time 12 months ago** | |
| **Quit attempt** | During the PAST 12 MONTHS, have you TRIED to QUIT smoking COMPLETELY? |
| **Smoked more than 12 days in the past 30-days around this time 12 months ago** | |
| **Quit attempt** | During the past 12 months, have you stopped smoking for one day or longer BECAUSE YOU WERE TRYING TO QUIT SMOKING? |
| **Current Former smokers** | |
| **Time since quitting** | About how long has it been since you COMPLETELY quit smoking cigarettes? |
|  | Enter Unit Reported. |

**Figure S1.** Flow chart of exclusions for the study sample

Self-respondents and 18 years or older (N=137,471)

Non-established smoking (N=91,442)

Now smoke “not at all” (N=29,189)

Has been less than or equal to 1 year since completely quitting smoking cigarettes (N=2,156)

Has been more than 1 year since completely quitting smoking cigarettes (N=27,033)

Unknown smoking frequency 12 months ago (N=38)

Smoked “every day” or “some days” 12 months ago (N=1,679)

Known smoking frequency 12 months ago (N=1,641)

Responded “yes” or “no” to have you ever tried to quit smoking? (N=14,950)

Unknown response to have you ever tried to quit smoking? (N=99)

Smoked “every day” or “some days” 12 months ago (N=15,049)

Not smoked 12 months ago (N=1,521)

Now smoke every day or some days (N=16,570)

Unknown current smoking status (N=270)

Known current smoking status (N=45,759)

Established smoking (N=46,029)

Total currently smoke (N=14,950)

Total formerly smoked (N=1,641)

Reported not smoking 12 months ago (N=477)

**Table S2.** Characteristics of e-cigarette use groups among people who smoked cigarettes 12 months ago. E-cigarette use categories: 1) Non e-cig user, 2) Current e-cig user with non-flavored or exclusive tobacco flavored, 3) Current e-cig user with flavors

| Variable | Categories | Quit attempt model sample | | | | |
| --- | --- | --- | --- | --- | --- | --- |
|  |  | Non e-cig user | Current e-cig user with non-flavored or exclusive tobacco-flavored | p-value  vs. non e-cig user | Current e-cig user  with flavors | p-value  vs. non e-cig user |
| Gender | Male | 50.0% | 46.8% | 0.228 | 46.4% | **0.037** |
|  | Female | 50.0% | 53.2% |  | 53.6% |  |
| Age | 18-21 | 1.4% | 2.0% | 0.462 | 6.5% | **<0.001** |
|  | 22-25 | 3.7% | 4.1% |  | 11.2% |  |
|  | 26-29 | 5.9% | 7.6% |  | 13.7% |  |
|  | 30-34 | 9.4% | 7.6% |  | 15.1% |  |
|  | 35-44 | 18.6% | 16.8% |  | 21.3% |  |
|  | 45-64 | 43.6% | 45.4% |  | 26.5% |  |
|  | 65+ | 17.4% | 16.6% |  | 5.7% |  |
| Race | White | 82.5% | 90.7% | **<0.001** | 88.2% | **<0.001** |
|  | Black | 11.2% | 3.4% |  | 4.9% |  |
|  | Asian | 2.0% | 2.2% |  | 2.5% |  |
|  | Other Races | 4.3% | 3.7% |  | 4.4% |  |
| Hispanic | Hispanic | 7.0% | 5.6% | 0.318 | 7.8% | 0.417 |
|  | Non-Hispanic | 93.0% | 94.4% |  | 92.2% |  |
| Education | Less than 12 years | 14.5% | 12.7% | 0.054 | 8.8% | **<0.001** |
|  | High school degree | 39.2% | 34.1% |  | 38.7% |  |
|  | Some college, no degree | 21.3% | 23.9% |  | 24.6% |  |
|  | College degree or higher | 25.0% | 29.3% |  | 27.9% |  |
| Family income | $0-$19999 | 24.9% | 22.9% | **0.037** | 18.3% | **<0.001** |
|  | $20000-$39999 | 27.0% | 22.4% |  | 26.7% |  |
|  | $40000-$74999 | 27.1% | 28.8% |  | 31.6% |  |
|  | $75000 or more | 21.0% | 25.9% |  | 23.4% |  |
| Marital status | Never Married | 27.1% | 26.3% | 0.393 | 44.0% | **<0.001** |
|  | Married Present | 36.1% | 33.2% |  | 28.7% |  |
|  | Married-Spouse Absent | 1.7% | 1.5% |  | 0.8% |  |
|  | Widowed/Divorced/Separated | 35.1% | 39.0% |  | 26.4% |  |
| Employment | Employed | 56.5% | 57.6% | 0.719 | 70.9% | **<0.001** |
|  | Not in labor force or unemployed | 43.5% | 42.4% |  | 29.1% |  |
| Metropolitan status | Metropolitan | 71.6% | 78.3% | **0.004** | 76.3% | **0.002** |
|  | Non-metropolitan | 28.4% | 21.7% |  | 23.7% |  |
| Indoor workers | No | 64.0% | 61.7% | 0.371 | 52.0% | **<0.001** |
|  | Yes | 36.0% | 38.3% |  | 48.0% |  |
| Cigarettes per day 12 months ago | 1--4 | 19.2% | 19.0% | **0.002** | 21.4% | **0.010** |
|  | 5--14 | 35.7% | 31.2% |  | 38.2% |  |
|  | 15--24 | 34.4% | 36.3% |  | 31.4% |  |
|  | 25+ | 7.5% | 12.0% |  | 7.4% |  |
|  | Unknown CPD | 3.2% | 1.5% |  | 1.6% |  |
| Smoking frequency | Everyday smokers 12 months ago | 80.1% | 79.0% | 0.646 | 75.5% | **0.001** |
|  | Someday smokers 12 months ago | 19.9% | 21.0% |  | 24.5% |  |

**Table S3.** Characteristics of e-cigarette use groups among people who smoked 12 months ago. E-cigarette use categories: 1) Non e-cig user, 2) Current e-cig user with non-flavored or exclusive tobacco flavored, 3) Current e-cig user with menthol or mint flavor, 4) Current e-cig user with other flavors

| Variable | Categories | Quit attempt model sample | | | | | | |
| --- | --- | --- | --- | --- | --- | --- | --- | --- |
|  |  | Non e-cig user | Current e-cig user with non-flavored or exclusive tobacco-flavored | p-value  vs. non e-cig user | Current e-cig user with menthol or mint flavor | p-value  vs. non e-cig user | Current e-cig user with other flavors | p-value  vs. non e-cig user |
| Gender | Male | 50.0% | 46.8% | 0.228 | 50.2% | 0.994 | 44.7% | **0.009** |
|  | Female | 50.0% | 53.2% |  | 49.8% |  | 55.3% |  |
| Age | 18-21 | 1.4% | 2.0% | 0.462 | 7.9% | **<0.001** | 25.8% | **<0.001** |
|  | 22-25 | 3.7% | 4.1% |  | 9.2% |  | 5.8% |  |
|  | 26-29 | 5.9% | 7.6% |  | 12.9% |  | 12.1% |  |
|  | 30-34 | 9.4% | 7.6% |  | 15.8% |  | 14.1% |  |
|  | 35-44 | 18.6% | 16.8% |  | 19.1% |  | 14.7% |  |
|  | 45-64 | 43.6% | 45.4% |  | 28.1% |  | 22.3% |  |
|  | 65+ | 17.4% | 16.6% |  | 6.9% |  | 5.1% |  |
| Race | White | 82.5% | 90.7% | **<0.001** | 82.2% | 0.096 | 90.9% | **<0.001** |
|  | Black | 11.2% | 3.4% |  | 9.2% |  | 2.9% |  |
|  | Asian | 2.0% | 2.2% |  | 4.0% |  | 1.8% |  |
|  | Other Races | 4.3% | 3.7% |  | 4.6% |  | 4.3% |  |
| Hispanic | Hispanic | 7.0% | 5.6% | 0.318 | 7.9% | 0.616 | 7.7% | 0.563 |
|  | Non-Hispanic | 93.0% | 94.4% |  | 92.1% |  | 92.3% |  |
| Education | Less than 12 years | 14.5% | 12.7% | 0.054 | 8.3% | **0.003** | 9.1% | **0.001** |
|  | High school degree | 39.2% | 34.1% |  | 38.0% |  | 39.0% |  |
|  | Some college, no degree | 21.3% | 23.9% |  | 27.7% |  | 23.2% |  |
|  | College degree or higher | 25.0% | 29.3% |  | 26.1% |  | 28.7% |  |
| Family income | $0-$19999 | 24.9% | 22.9% | **0.037** | 18.2% | **0.023** | 18.4% | **0.001** |
|  | $20000-$39999 | 27.0% | 22.4% |  | 25.7% |  | 27.2% |  |
|  | $40000-$74999 | 27.1% | 28.8% |  | 31.4% |  | 31.6% |  |
|  | $75000 or more | 21.0% | 25.9% |  | 24.8% |  | 22.7% |  |
| Marital status | Never Married | 27.1% | 26.3% | 0.393 | 47.2% | **<0.001** | 42.5% | **<0.001** |
|  | Married Present | 36.1% | 33.2% |  | 25.4% |  | 30.3% |  |
|  | Married-Spouse Absent | 1.7% | 1.5% |  | 0.7% |  | 0.9% |  |
|  | Widowed/Divorced/Separated | 35.1% | 39.0% |  | 26.7% |  | 26.3% |  |
| Employment | Employed | 56.5% | 57.6% | 0.719 | 67.7% | **<0.001** | 72.4% | **<0.001** |
|  | Not in labor force or unemployed | 43.5% | 42.4% |  | 32.3% |  | 27.6% |  |
| Metropolitan status | Metropolitan | 71.6% | 78.3% | **0.004** | 77.6% | **0.027** | 75.7% | **0.024** |
|  | Non-metropolitan | 28.4% | 21.7% |  | 22.4% |  | 24.3% |  |
| Indoor workers | No | 64.0% | 61.7% | 0.371 | 53.5% | **<0.001** | 51.3% | **<0.001** |
|  | Yes | 36.0% | 38.3% |  | 46.5% |  | 48.7% |  |
| Cigarettes per day 12 months ago | 1--4 | 19.2% | 19.0% | **0.002** | 21.5% | 0.071 | 21.4% | 0.179 |
|  | 5--14 | 35.7% | 31.2% |  | 40.3% |  | 37.2% |  |
|  | 15--24 | 34.4% | 36.3% |  | 30.0% |  | 32.1% |  |
|  | 25+ | 7.5% | 12.0% |  | 7.3% |  | 7.5% |  |
|  | Unknown CPD | 3.2% | 1.5% |  | 1.0% |  | 1.8% |  |
| Smoking frequency | Everyday smokers 12 months ago | 80.1% | 79.0% | 0.646 | 75.6% | 0.063 | 75.4% | **0.004** |
|  | Someday smokers 12 months ago | 19.9% | 21.0% |  | 24.4% |  | 24.6% |  |

**Table S4.** Characteristics of e-cigarette use groups among people who smoked 12 months ago and made at least one quit attempt. E-cigarette use categories: 1) Non e-cig user, 2) Current e-cig user with non-flavored or exclusive tobacco flavored, 3) Current e-cig user with flavors

| Variable | Categories | Quit success model sample | | | | |
| --- | --- | --- | --- | --- | --- | --- |
|  |  | Non e-cig user | Current e-cig user with non-flavored or exclusive tobacco-flavored | p-value  vs. non e-cig user | Current e-cig user  with flavors | p-value  vs. non e-cig user |
| Gender | Male | 52.3% | 49.1% | 0.382 | 46.2% | **0.005** |
|  | Female | 47.7% | 50.9% |  | 53.8% |  |
| Age | 18-21 | 1.7% | 2.2% | 0.474 | 5.9% | **<0.001** |
|  | 22-25 | 5.0% | 4.8% |  | 10.0% |  |
|  | 26-29 | 7.3% | 9.6% |  | 14.4% |  |
|  | 30-34 | 10.3% | 7.0% |  | 15.7% |  |
|  | 35-44 | 18.3% | 16.7% |  | 20.8% |  |
|  | 45-64 | 41.2% | 44.7% |  | 27.2% |  |
|  | 65+ | 16.2% | 14.9% |  | 5.9% |  |
| Race | White | 80.0% | 92.5% | **<0.001** | 87.6% | **<0.001** |
|  | Black | 13.0% | 2.6% |  | 5.2% |  |
|  | Asian | 2.0% | 1.8% |  | 2.9% |  |
|  | Other Races | 5.0% | 3.1% |  | 4.2% |  |
| Hispanic | Hispanic | 7.6% | 7.9% | 0.980 | 8.1% | 0.723 |
|  | Non-Hispanic | 92.4% | 92.1% |  | 91.9% |  |
| Education | Less than 12 years | 13.8% | 11.8% | 0.210 | 9.3% | **0.013** |
|  | High school degree | 37.3% | 35.1% |  | 36.7% |  |
|  | Some college, no degree | 22.7% | 20.6% |  | 24.7% |  |
|  | College degree or higher | 26.2% | 32.5% |  | 29.3% |  |
| Family income | $0-$19999 | 26.6% | 23.7% | 0.158 | 19.6% | **0.001** |
|  | $20000-$39999 | 25.6% | 21.1% |  | 25.9% |  |
|  | $40000-$74999 | 26.5% | 31.6% |  | 31.3% |  |
|  | $75000 or more | 21.3% | 23.7% |  | 23.2% |  |
| Marital status | Never Married | 29.1% | 25.9% | 0.183 | 42.8% | **<0.001** |
|  | Married Present | 34.4% | 33.8% |  | 27.9% |  |
|  | Married-Spouse Absent | 1.8% | 0.4% |  | 0.3% |  |
|  | Widowed/Divorced/Separated | 34.7% | 39.9% |  | 28.9% |  |
| Employment | Employed | 56.6% | 56.1% | 0.948 | 70.7% | **<0.001** |
|  | Not in labor force or unemployed | 43.4% | 43.9% |  | 29.3% |  |
| Metropolitan status | Metropolitan | 73.1% | 81.6% | **0.006** | 76.6% | 0.071 |
|  | Non-metropolitan | 26.9% | 18.4% |  | 23.4% |  |
| Indoor workers | No | 62.9% | 60.5% | 0.504 | 50.3% | **<0.001** |
|  | Yes | 37.1% | 39.5% |  | 49.7% |  |
| Cigarettes per day 12 months ago | 1--4 | 24.9% | 23.2% | 0.110 | 23.7% | 0.395 |
|  | 5--14 | 37.3% | 34.2% |  | 37.2% |  |
|  | 15--24 | 29.1% | 32.0% |  | 31.1% |  |
|  | 25+ | 5.4% | 8.8% |  | 5.9% |  |
|  | Unknown CPD | 3.3% | 1.8% |  | 2.0% |  |
| Smoking frequency | Everyday smokers 12 months ago | 72.3% | 72.4% | 1.000 | 72.1% | 0.947 |
|  | Someday smokers 12 months ago | 27.7% | 27.6% |  | 27.9% |  |

**Table S5.** Characteristics of e-cigarette use groups among people who smoked 12 months ago and made at least one quit attempt. E-cigarette use categories: 1) Non e-cig user, 2) Current e-cig user with non-flavored or exclusive tobacco flavored, 3) Current e-cig user with menthol or mint flavor, 4) Current e-cig user with other flavors

|  |  | Quit success model sample | | | | | | |
| --- | --- | --- | --- | --- | --- | --- | --- | --- |
|  |  | Non e-cig user | Current e-cig user with non-flavored or exclusive tobacco-flavored | p-value  vs. non e-cig user | Current e-cig user with menthol or mint flavor | p-value  vs. non e-cig user | Current e-cig user with other flavors | p-value  vs. non e-cig user |
| Gender | Male | 52.3% | 49.1% | 0.382 | 50.0% | 0.573 | 44.3% | **0.002** |
|  | Female | 47.7% | 50.9% |  | 50.0% |  | 55.7% |  |
| Age | 18-21 | 1.7% | 2.2% | 0.474 | 7.6% | **<0.001** | 5.1% | **<0.001** |
|  | 22-25 | 5.0% | 4.8% |  | 5.6% |  | 12.2% |  |
|  | 26-29 | 7.3% | 9.6% |  | 12.6% |  | 15.3% |  |
|  | 30-34 | 10.3% | 7.0% |  | 18.7% |  | 14.2% |  |
|  | 35-44 | 18.3% | 16.7% |  | 20.2% |  | 21.1% |  |
|  | 45-64 | 41.2% | 44.7% |  | 27.8% |  | 27.0% |  |
|  | 65+ | 16.2% | 14.9% |  | 7.6% |  | 5.1% |  |
| Race | White | 80.0% | 92.5% | **<0.001** | 81.3% | 0.151 | 90.8% | **<0.001** |
|  | Black | 13.0% | 2.6% |  | 10.1% |  | 2.8% |  |
|  | Asian | 2.0% | 1.8% |  | 4.0% |  | 2.3% |  |
|  | Other Races | 5.0% | 3.1% |  | 4.5% |  | 4.1% |  |
| Hispanic | Hispanic | 7.6% | 7.9% | 0.980 | 8.6% | 0.715 | 7.9% | 0.925 |
|  | Non-Hispanic | 92.4% | 92.1% |  | 91.4% |  | 92.1% |  |
| Education | Less than 12 years | 13.8% | 11.8% | 0.210 | 9.6% | 0.190 | 9.2% | 0.054 |
|  | High school degree | 37.3% | 35.1% |  | 34.8% |  | 37.7% |  |
|  | Some college, no degree | 22.7% | 20.6% |  | 27.3% |  | 23.4% |  |
|  | College degree or higher | 26.2% | 32.5% |  | 28.3% |  | 29.8% |  |
| Family income | $0-$19999 | 26.6% | 23.7% | 0.158 | 18.7% | **0.048** | 20.1% | **0.019** |
|  | $20000-$39999 | 25.6% | 21.1% |  | 24.7% |  | 26.5% |  |
|  | $40000-$74999 | 26.5% | 31.6% |  | 30.3% |  | 31.8% |  |
|  | $75000 or more | 21.3% | 23.7% |  | 26.3% |  | 21.6% |  |
| Marital status | Never Married | 29.1% | 25.9% | 0.183 | 44.4% | **<0.001** | 42.0% | **<0.001** |
|  | Married Present | 34.4% | 33.8% |  | 26.3% |  | 28.8% |  |
|  | Married-Spouse Absent | 1.8% | 0.4% |  | 0.0% |  | 0.5% |  |
|  | Widowed/Divorced/Separated | 34.7% | 39.9% |  | 29.3% |  | 28.8% |  |
| Employment | Employed | 56.6% | 56.1% | 0.948 | 67.2% | **0.004** | 72.5% | **<0.001** |
|  | Not in labor force or unemployed | 43.4% | 43.9% |  | 32.8% |  | 27.5% |  |
| Metropolitan status | Metropolitan | 73.1% | 81.6% | **0.006** | 79.3% | 0.064 | 75.3% | 0.369 |
|  | Non-metropolitan | 26.9% | 18.4% |  | 20.7% |  | 24.7% |  |
| Indoor workers | No | 62.9% | 60.5% | 0.504 | 53.0% | **0.006** | 48.9% | **<0.001** |
|  | Yes | 37.1% | 39.5% |  | 47.0% |  | 51.1% |  |
| Cigarettes per day 12 months ago | 1--4 | 24.9% | 23.2% | 0.110 | 26.3% | 0.589 | 22.4% | 0.566 |
|  | 5--14 | 37.3% | 34.2% |  | 35.4% |  | 38.2% |  |
|  | 15--24 | 29.1% | 32.0% |  | 30.3% |  | 31.6% |  |
|  | 25+ | 5.4% | 8.8% |  | 6.6% |  | 5.6% |  |
|  | Unknown CPD | 3.3% | 1.8% |  | 1.5% |  | 2.3% |  |
| Smoking frequency | Everyday smokers 12 months ago | 72.3% | 72.4% | 1.000 | 70.7% | 0.680 | 72.8% | 0.886 |
|  | Someday smokers 12 months ago | 27.7% | 27.6% |  | 29.3% |  | 27.2% |  |
